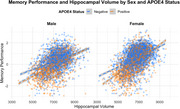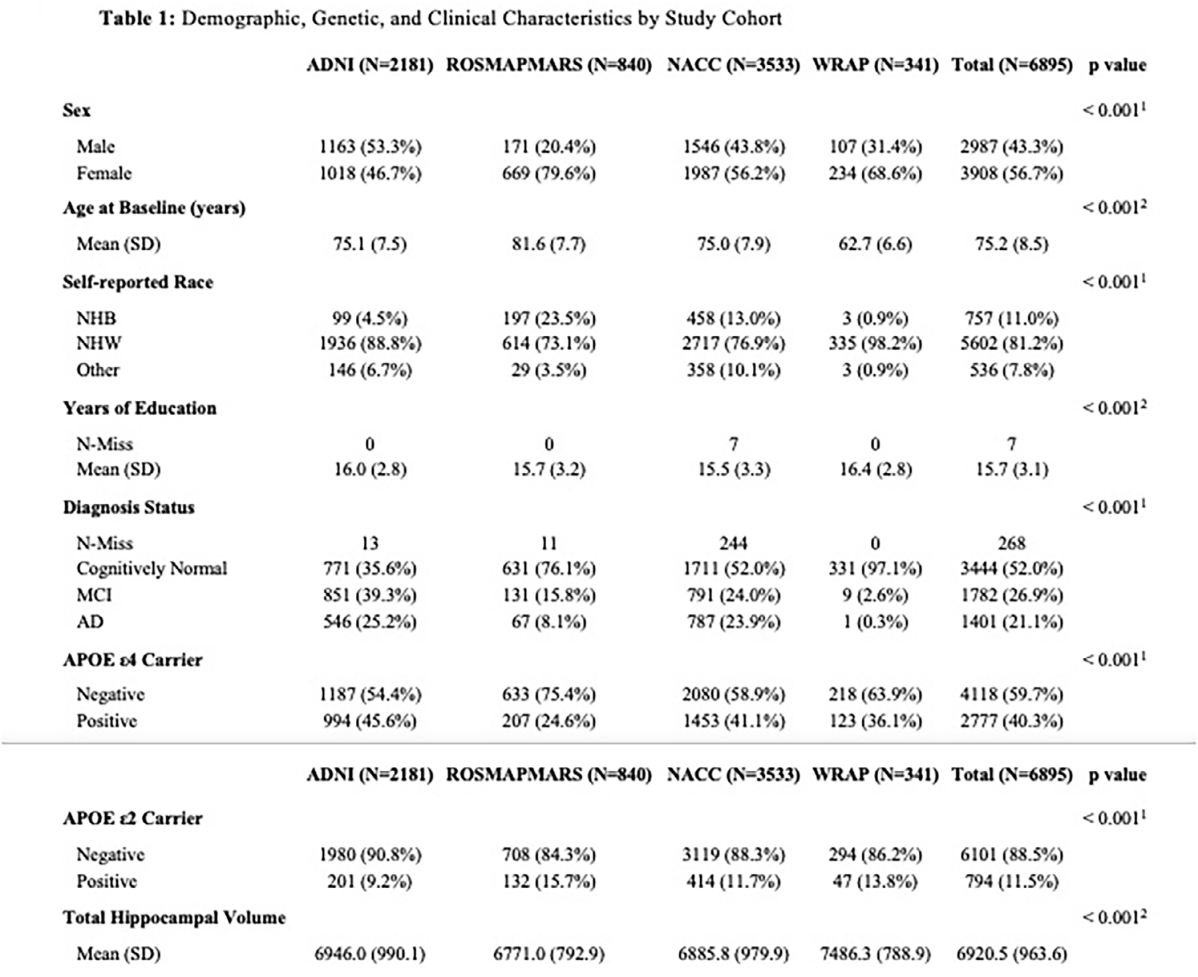# Sex and APOE Genotype Differentially Modify the Association Between Hippocampal Volume and Memory Performance

**DOI:** 10.1002/alz70855_104990

**Published:** 2025-12-24

**Authors:** Abel Belachew, Sarah Biber, Walter W. Kukull, Shubhabrata Mukherjee, Paul K Crane, Jesse Mez, Seo‐Eun Choi, Brandon Klinedinst, Michael L. Lee, Phoebe Scollard, Shannon Risacher, Meichen Yu, Christos Davatzikos, Haochang Shou, Guray Erus, Yuhan Cui, Adam C. Naj, Li‐San Wang, Jonathan L Haines, Margaret Pericak‐Vance, Richard Mayeux, Alaina Durant, Timothy J. Hohman, Derek Archer, Logan Dumitrescu

**Affiliations:** ^1^ Vanderbilt Memory & Alzheimer's Center, Vanderbilt University Medical Center, Nashville, TN, USA; ^2^ Department of Medicine, University of Washington, Seattle, WA, USA; ^3^ Department of General Internal Medicine, University of Washington School of Medicine, Seattle, WA, USA; ^4^ Department of Neurology, Boston University Chobanian & Avedisian School of Medicin, Boston, MA, USA; ^5^ School of Medicine, Indiana University, Indianapolis, IN, USA; ^6^ Perelman School of Medicine, University of Pennsylvania, Philadelphia, PA, USA; ^7^ University of Pennsylvania Perelman School of Medicine, Philadelphia, PA, USA; ^8^ School of Medicine, Case Western Reserve University, Cleveland, OH, USA; ^9^ Miller School of Medicine, University of Miami, Coral Gables, FL, USA; ^10^ Department of Neurology and the Taub Institute for the Study of Alzheimer's Disease and the Aging Brain, Columbia University Irving Medical Center, New York, NY, USA; ^11^ Vanderbilt University Medical Center, Nashville, TN, USA; ^12^ Vanderbilt Genetics Institute, Vanderbilt University Medical Center, Nashville, TN, USA; ^13^ Vanderbilt Memory and Alzheimer's Center, Vanderbilt University School of Medicine, Nashville, TN, USA

## Abstract

**Background:**

The hippocampus, vital for memory processing, is one of the first regions affected by Alzheimer's disease (AD). *APOE‐ε4* (risk) and *APOE*‐ε2 (protective) alleles are key genetic drivers of AD risk and cognitive decline. This study aimed to assess the modifying effect of the *APOE* genotype on the association between hippocampal volume and memory performance. Furthermore, we considered the modifying effects of sex, self‐reported race, and clinical diagnosis.

**Methods:**

Data were obtained from 6,895 participants (mean age at baseline=75.2 years; 21% AD, 43% male, 81% non‐Hispanic white (NHW), 12% *APOE‐ε2* carriers, and 40% *APOE‐ε4* carriers) from four cohorts of aging and AD: ADNI, NACC, ROS/MAP/MARS, and WRAP. MRI data, processed with deep learning MUSE, included left and right hippocampal volumes, harmonized for batch differences using Longitudinal Combat. Memory composite scores were harmonized across cohorts using latent variable modeling. Linear regression on baseline memory assessed two‐way interactions between total hippocampal volume×sex, total hippocampal volume×*APOE* (modeled dominantly), and three‐way interactions that included sex, race, or diagnosis. Mixed effects regression models assessed these interactions on memory trajectories and included fixed and random effects for intercept and the slope (years from baseline).

**Results:**

The association between hippocampal volume and memory performance is stronger in females than in males, whereby females with larger hippocampi outperform males with larger hippocampi (*p* = 7.47×10^‐13^; R^2^=0.007; Figure 1). *APOE* haplotype also interacted with hippocampal volume on baseline memory, whereby the association is stronger in *ε4*(*p* = 6.07×10^−13^; R^2^=0.004) carriers and attenuated in *ε2* carriers (*p* = 3.22×10^−5^; R^2^=0.001), particularly among individuals with mild cognitive impairment (*p* = 0.048; b=5.41×10^‐5^). Notably, *APOE* interactions with hippocampal volume were consistent across NHW and non‐Hispanic black (NHB) participants. In longitudinal analyses, we found a significant three‐way interaction between *APOE‐ε2*, AD diagnosis, and hippocampal volume on memory (*p* = 0.0001; R^2^c=0.8; R^2^m=0.5), driven by a stronger interaction among participants with AD (*p* = 0.036; R^2^c=0.5; R^2^m=0.04).

**Conclusion:**

While hippocampal volume is a potent predictor of memory performance, sex, and *APOE* modify this association with the most pronounced effects observed among *APOE*‐ε4 carriers, women, and participants with AD. These results emphasize the importance of considering genetic, clinical, and demographic factors in AD research.